# Development and deployment of a high-density linkage map identified quantitative trait loci for plant height in peanut (*Arachis hypogaea* L.)

**DOI:** 10.1038/srep39478

**Published:** 2016-12-20

**Authors:** Li Huang, Xiaoping Ren, Bei Wu, Xinping Li, Weigang Chen, Xiaojing Zhou, Yuning Chen, Manish K. Pandey, Yongqing Jiao, Huaiyong Luo, Yong Lei, Rajeev K. Varshney, Boshou Liao, Huifang Jiang

**Affiliations:** 1 Key Laboratory of Biology and Genetic Improvement of Oil Crops, Ministry of Agriculture, Oil Crops Research Institute of the Chinese Academy of Agricultural Sciences, Wuhan, Hubei, China; 2 International Crops Research Institute for the Semi-Arid Tropics (ICRISAT), Hyderabad, India

## Abstract

Plant height is one of the most important architecture traits in crop plants. In peanut, the genetic basis of plant height remains ambiguous. In this context, we genotyped a recombinant inbred line (RIL) population with 140 individuals developed from a cross between two peanut varieties varying in plant height, Zhonghua 10 and ICG 12625. Genotyping data was generated for 1,175 SSR and 42 transposon polymorphic markers and a high-density genetic linkage map was constructed with 1,219 mapped loci covering total map length of 2,038.75 cM i.e., accounted for nearly 80% of the peanut genome. Quantitative trait locus (QTL) analysis using genotyping and phenotyping data for three environments identified 8 negative-effect QTLs and 10 positive-effect QTLs for plant height. Among these QTLs, 8 QTLs had a large contribution to plant height that explained ≥10% phenotypic variation. Two major-effect consensus QTLs namely *cqPHA4a* and *cqPHA4b* were identified with stable performance across three environments. Further, the allelic recombination of detected QTLs proved the existence of the phenomenon of transgressive segregation for plant height in the RIL population. Therefore, this study not only successfully reported a high-density genetic linkage map of peanut and identified genomic region controlling plant height but also opens opportunities for further gene discovery and molecular breeding for plant height in peanut.

Plant architecture plays an extremely important role for overall yield, economic value and harvest index due to its significant effect on light interception and photosynthesis^1^,^2^. Tremendous breeding efforts were made focusing on plant architecture modifications to improve crop adaptability to different environments and increase harvest index^3^,^4^. The global community has already witnessed the impact of breeding semi-dwarf or dwarf wheat and rice cultivars leading to “green revolution” which dramatically increased cereal production. Plant height is an important and highly heritable component of plant architecture and has significant effect on biomass yield and stalk lodging^5^. Because of their agronomic importance, different genes of dwarf mutants were isolated in rice^4^,^6^,^7^,^8^, maize^9^,^10^,^11^, and wheat^3^,^12^,^13^ and were found to be involved in biosynthesis or signaling pathways of phytohormones, such as gibberellin (GA) and brasinosteroid (BR)^5^,^6^,^7^,^8^,^10^.

Cultivated peanut (*Arachis hypogaea* L.) is an important oil crop in the world and is widely cultivated in tropical and sub-tropical regions. In addition to food and an edible oil for human consumption, this crop also serves as a good source of fodder for cattle. Cultivated peanut is an allotetraploid (AABB, 2n = 4x = 40) crop formed through the natural hybridization of its two progenitor diploids, *A. duranensis* (AA, 2n = 2x = 20) and *A. ipaensis* (BB, 2n = 2x = 20)^14^,^15^. Positive correlations were revealed between plant height and yield-related traits, such as pod length, pod width, hundred pod weight, seed length, seed width and hundred seed weight^16^ in Chinese peanut mini-core collection. Majority of the cultivated peanuts in China and many other countries belong to erect/bunch type, and the long height of peanut crop could lead to lodging. Currently, mechanization of harvesting which could save labor cost and increase working efficiency is widely applied in peanut. If the plant height of peanut is too dwarf or too high, the combine harvesters could miss some pods or make some peanut straw and pods mixed together. Therefore, plant height has become one of the increasingly important agronomic trait in peanut breeding. Understanding the genetic basis of plant height is conducive to enhance resistance to lodging, increase peanut yield and improve efficiency of mechanized harvesting.

Quantitative trait locus (QTL) analysis is one of the major trait mapping approach to identifying genomic loci that control agronomic traits in crops. Simple sequence repeat (SSR) markers have been the choice of genetic markers and were used for developing genetic maps for cultivated peanuts which in turn facilitated several QTL mapping studies in peanut using F_2_ and recombinant inbred lines (RIL) populations. Strikingly, many of these QTLs were detected for drought tolerance^17^,^18^,^19^, resistance to biotic stresses such as late leaf spot, rust and bacterial wilt resistance^20^,^21^,^22^, pod- and seed-related traits^23^,^24^,^25^ and quality traits^23^,^26^,^27^,^28^. However, only few studies focused on the plant height in peanut using F_2_ mapping populations^23^,^25^. Three QTLs for plant height with 4.8~19.2% phenotypic variation explained (PVE) were identified in LG04.2, LG05.2 and LG06.2 using 94 F_2_ lines in one year^25^. In our previous study, 3 QTLs for plant height with relatively large genetic distance ranging from 8 to 17 cM were identified based on an F_2_ mapping population^23^. The resolution of the above mentioned QTLs for plant height was relatively low due to the limited numbers of markers and the lack of phenotypic evaluation in multiple environments.

In this study, the phenotypic data of plant height across three environments were collected for a RIL (F_6_ generation) population from a cross between two peanut varieties, Zhonghua 10 (maternal parent) and ICG 12625 (paternal parent). The present study reports the development of high-density genetic linkage map for cultivated peanut; getting insights on the genetic basis of plant height in peanut and genome-wide identification of QTLs controlling plant height in peanut using the RIL population.

## Results

### Phenotypic variation of plant height

**Figure 1:**
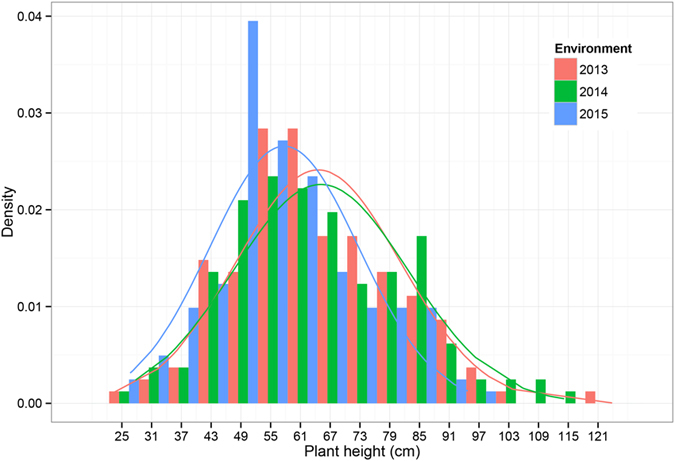
Phenotypic distribution of plant height in the peanut RIL population.

Phenotypic evaluation of two parental genotypes and RILs for three years (2013~2015) showed significant variation for plant height across the environments. Large phenotypic variation of plant height was observed between the RIL parents as well as in RIL population (Table 1). The plant height of maternal parent, Zhonghua 10, varied from 42.4 to 47.3 cm while the plant height of paternal parent, ICG 12625 (PI497597), varied from 62.1 to 73.9 cm in the three environments. The RILs showed high phenotypic variation for plant height ranging from 24.6~125.4 cm, 29.3~115.7 cm and 28.2~96.3 cm during 2013, 2014 and 2015, respectively. The plant height in the RIL population distributed normally in 2013 (*P* = 0.28) with near normal distribution during 2014 and 2015 (*P* = 0.01 and *P* = 0.02; Fig. 1). The broad-sense heritability of plant height was estimated to be relatively high (91.4%) indicating strong control by genetic factors and less environmental effects. Two-way analysis of variance (ANOVA) also revealed that genetic, environmental effects and genotype by environment interaction significantly influenced plant height (Table 2).

### Marker polymorphism and genetic map construction

**Figure 2:**
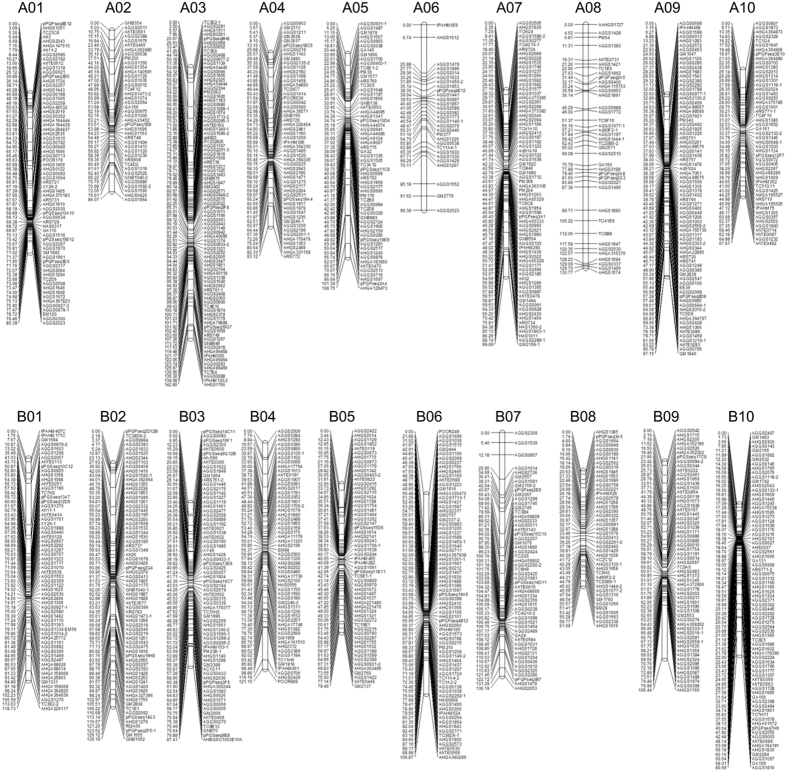
Genetic map in an F_6_ population derived from a cross by Zhonghua 10 and ICG 12625.

Screening of a set of 7,641 SSR and 427 transposon markers on two parental genotypes of the RIL population resulted in identification of 1,217 (1,175 SSRs and 42 transposon) polymorphic markers. Genotyping of these polymorphic markers amplified 1,272 loci, of which 1,163 markers had one locus for each marker, 53 markers had two loci for each marker and one marker had three loci. Subsequently, a high-density genetic map containing 1,219 loci was constructed covering a total map length of 2,038.75 cM of the peanut genome with an average map density of 1.67 cM per loci (Fig. 2, Table 3). All the loci were assigned to 20 linkage groups designated as A01-A10 for A subgenome and B01-B10 for B subgenome by aligning the markers to the previously published map^29^. There were 583 loci for the A subgenome and 636 loci for the B subgenome with the map length of 1,010.95 and 1,027.80 cM, respectively. The length of linkage groups ranged from 61.69 (B08) to 142.80 (A03) cM. The marker density among linkage groups ranged from 1.05 (B10) to 3.43 (A06) cM/loci. Chi-square (χ^2^) analysis revealed significant segregation distortion (*P* < 0.05) for 152 loci (12.5%), of which 102 and 50 SSR loci skewed towards Zhonghua 10 and ICG 12625, respectively (Table 3). The skewed loci on linkage groups A03, A06, A09, B02, B07, B09 and B10 favored the parent ‘Zhonghua 10’ allele, and linkage groups A02 and B01 contained loci favoring the parent ‘ICG 12625’ allele. In addition, no more than five skewed loci were mapped on linkage groups A01, A04, A05, A10, B02, B03, B05 and B08 (Supplementary Dataset 1).

### QTL mapping and meta-analysis for plant height

**Figure 3:**
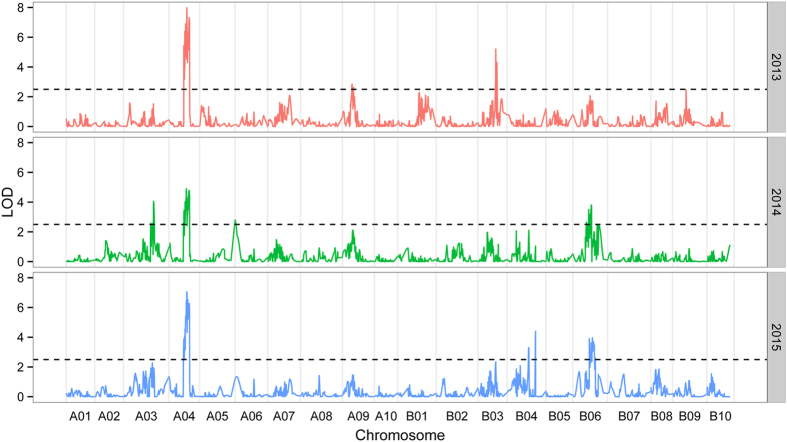
QTL overviews of plant height across three environments.

Genome-wide QTL analysis was performed using the high-density genetic map and the phenotypic data of plant height obtained from the RILs during 2013, 2014 and 2015 in Wuhan. For plant height, a total of 18 QTLs were identified in three environments that explained 4.85~20.52% of the phenotypic variation (Fig. 3, Table 4). Five major QTLs namely *qPHA4.1a, qPHA4.1b, qPHA4.1c, qPHA4.1d*, and *qPHB3.1* and one minor QTL namely *qPHA9.1* were identified in 2013 environment, which explained 6.04~20.52% of the phenotypic variation. In 2014 environment, one major QTL, *qPHA4.2b* and six minor QTLs, *qPHA3.2a, qPHA3.2b, qPHA4.2a, qPHB6.2a, qPHB6.2b*, and *qPHB6.2c* were identified with a range of 4.85~10.84% PVE. In 2015 environment, two major QTLs, *qPHA4.3* and *qPHB4.3a*, and three minor QTLs, *qPHB4.3b, qPHB6.3a* and *qPHB6.3b*, were detected with a range of 5.23~14.47% phenotypic variation. A total of 8 QTLs explaining more than 10% of PV were identified in three environments. However, all identified QTLs jointly explained 53.3~65.5% of plant height variation based on multiple linear regressions. Epistasis analysis revealed two nominal interactions of pair-wise QTLs (*P* < 0.02 and *P* < 0.05), *qPHA4.3* and *qPHB4.3b, qPHB6.2b* and *qPHB6.2c*, which contributed little to plant height variation (*R*^2^ = 0.059 and *R*^2^ = 0.061), respectively (Supplementary Dataset 2). Thus, we observed that the epistasis is significantly less important relative to additive effect in present study.

Of the18 QTLs identified in three environments, 7 QTLs (38.9%) were detected on LG A04 with 9.45~20.52% PVE, and 5 QTLs (27.8%) were detected on LG B06 with 5.00~7.77% PVE, suggesting that there were QTL clusters on LG A04 and B06. It is worth mentioning that four major QTLs detected on LG A04 in 2013 environment collectively explained as much as 84.98% PVE and had relatively high LOD values with a range of 6.39~7.98. These results indicated that LG A04 is rich in genes controlling plant height. In addition, 7 QTLs detected on LG A04 and one QTL detected on LG B03 in three environments had negative additive genetic effects (Fig. S1), which revealed maternal parent Zhonghua 10 as the source of alleles improving the plant height. The remaining 10 QTLs had positive additive genetic effects, suggesting that the alleles of these QTLs for increased high plant height came from the paternal parent ICG 12625.

To further dissect the QTLs controlling plant height, we integrated summary of QTL information in multiple environments via meta-analysis. Of 18 QTLs of plant height, four QTLs were identified as the reproducible QTL in multiple environments, whose confidence intervals (CI) were overlapped with at least one QTL (Fig. 1). These sets of reproducible QTLs were subsequently integrated into consensus QTL using meta-analysis. Specifically, two reproducible QTLs, *qPHA4.1c* (CI: 61.5~62.7 cM) and *qPHA4.3* (CI: 61.9~62.6 cM), were integrated into a consensus QTL named *cqPHA4a* (CI: 61.6~62.2 cM). Another two QTLs, *qPHA4.1d* (70.1~71.5 cM) and *qPHA4.2b* (67.0~71.4 cM), were integrated into another consensus QTL named *cqPHA4b* (CI: 69.7~71.0 cM) (Tables 4 and 5). Comparing consensus QTL with their original QTLs improved the resolution by 2–3.4 fold, indicating the QTL meta-analysis was feasible to finely explore the QTL originally detected in multiple environments in the present study.

### Recombination of QTL and transgressive segregation in the RIL population

We observed the extensively phenotypic variations in the RIL population that exhibited a large-scale transgressive segregation for plant height (Fig. 1), almost accounting for half of all lines in the RIL population. There were 6 RILs with shorter plant height than Zhonghua 10 and 43 RILs taller than ICG 12625 in 2013, 16 RILs shorter than Zhonghua 10 and 62 RILs taller than ICG 12625 in 2014 and 18 RILs shorter than Zhonghua 10 and 54 RILs taller than ICG 12625 in 2015. The results suggested that the increasing-effect alleles for plant height may be resided in both the parents, which was congruent to the finding of the additive directions of QTLs detected in the present study (Fig. S1).

**Figure 4:**
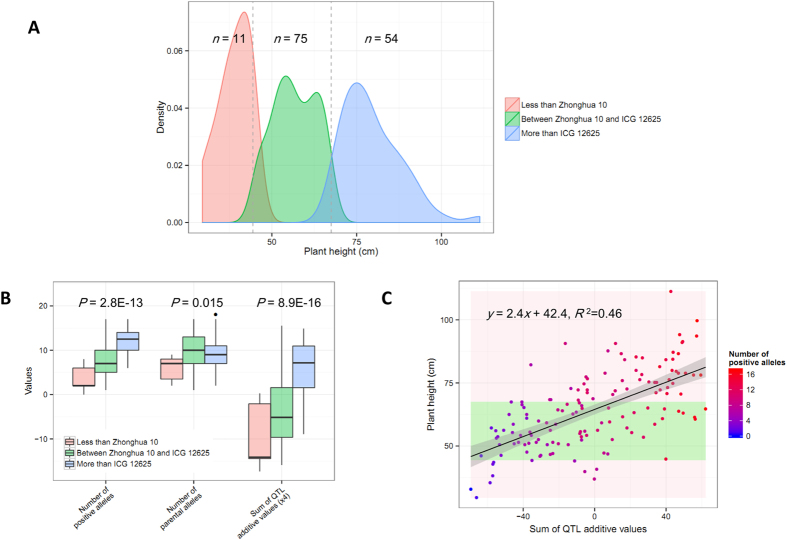
Transgressive segregation of plant height and allelic recombination in RIL population. (**A**) The distribution of plant height for lines belonging to the three groups. The lines from the group ‘less than Zhonghua 10’, ‘between Zhonghua 10 and ICG 12625’, and ‘more than ICG 12625’, were indicated by different colors, respectively. The dashed vertical lines indicated the phenotypic value of Zhonghua 10 (left) and ICG 12625 (right). (**B**) The difference of allele counts and additive values among three phenotypic groups. (**C**) The scatterplot of plant height against to total QTL additive values in the RIL population. The color gradient means the counts of positive alleles harbored in each RIL line.

To explore the genetic basis of the transgressive segregation of plant height, we evaluated the allelic recombination of QTLs in the RIL population. On the basis of the average plant height across three environments, the whole RIL population were divided into three PH-groups, named ‘Less than Zhonghua 10’ (*n* = 11), ‘Between Zhonghua 10 and ICG 12625’ (*n* = 75) and ‘More than ICG 12625’ (*n*= 54), respectively (Fig. 4A). The relationship between plant height and the compositions of QTL alleles for each PH-group was tested using ANOVA analysis. A nominal difference between three PH-groups was observed for the number of QTL parental alleles harbored in each line (*P* = 0.015), while the significantly differences were found for the number of positive alleles (*P* = 2.8E-13) and sum of additive values (*P* = 8.9E-16) for all QTLs in each lines, respectively (Fig. 4B). Additionally, the two statistics (i.e., the number of positive alleles and sum of additive values) demonstrated an identical trend of relationship with the plant height variations in the whole population, which could explain 46% PV for plant height in the population (Fig. 4C).

## Discussion

The construction of a genetic linkage map with optimum density is a prerequisite for conducting QTL analysis in the biparental population. More importantly, genome coverage level and marker density of the genetic map significantly impacts not only the sensitivity of QTL detection but also affects number of identified QTL^30^. The unavailability of optimum genomic resources especially SSR markers together with low level of DNA polymorphism in cultivated gene pool have hindered development of dense genetic maps for a long time in peanut^31^. Among marker types, SSRs gained wide acceptance in the scientific community because of their abundance and their ease of use for DNA fingerprinting, checking adulteration and impurity, genetic diversity, trait mapping and molecular breeding. In case of cultivated peanut, the first SSR-based genetic map was constructed using only 135 SSR markers, covering a total of 1,270.5 cM map distance which covers half of the peanut integrated consensus map^29^. In the past decade, thousands of SSR markers have been developed in peanut from complementary DNAs (cDNAs), SSR-enriched genomic DNA libraries, and BAC-ends^25^,^32^,^33^,^34^,^35^,^36^,^37^, which greatly accelerated the construction of genetic map and QTL analysis in peanut^21^,^23^,^24^,^25^,^27^,^28^,^29^,^38^,^39^,^40^. Since the polymorphism of SSRs in a specific population largely depended on the genetic diversity between the parents of an experimental population, several such efforts were required to make available large number of SSRs in the public domain for genetic and QTL mapping studies. In fact 2016 has been a great year because of the availability of genome sequence for both the subgenomes of the tetraploid peanut^15^,^41^, thereby initiating an era where large number of structural variations have been identified and can be used as genetic markers for generating high throughput genotyping data on genetic populations. Additionally, the previously studies on QTL analysis in peanut were based on either the limited SSRs (no more than 400)^19^,^21^,^27^,^28^,^39^ or the F_2_ mapping population^23^,^24^,^25^ posing difficulty in understanding the genetic basis of the complex traits whose phenotypes needed to be investigated repeatedly in multiple environments. In the present study, 1,175 SSR and 42 transposon polymorphic markers were used to genotype a peanut RIL population. Subsequently, we constructed a high-density linkage map with a total of 1,219 loci with the map length of 2,038.75 cM, covering nearly 80% of peanut genetic map represented by the integrated peanut map^29^. More than 85% of the 1,219 loci segregated in the population at the expected ratio of 1:1, which was significantly more than that of other studies^23^,^25^,^29^,^39^,^40^. The high proportion of mapped loci following Mendelian segregation has provided good quality and precise genetic map for conducting QTL analysis. Prior to this study, Shirasawa *et al*.^29^ constructed a linkage map with 1,469 loci of which majority were SSRs using mere 91 individuals of a RIL population. It is important to note that above mentioned population was developed from a cross between an elite and an artificial amphidiploid (*A. ipaensis* × *A. duranensis*)^4×^, and the population size was not big enough (n = 91) to conduct high resolution QTL detection i.e., too for complex traits. Therefore, it is worth mentioning here that the genetic map provided in our study is one of the highest-density maps of cultivated peanut in a permanent experimental population, which would not only give us the opportunity to deeply explore the genetic basis of agriculturally important traits but will also be beneficial to improve the draft sequence of the peanut tetraploid reference genome^15^.

Plant height is highly heritable trait due to accurate trait measurements leading to availability of reliable phenotyping data for conducting genetic analysis and selection^5^. Tremendous efforts have been made to identify QTLs or candidate genes for plant height in rice^4^,^6^,^7^,^8^, maize^9^,^10^,^11^, wheat^12^,^13^ and other crop species. However, limited efforts were made for doing genetic dissection and QTL discovery for plant height in peanut. Shirasawa *et al*. reported three QTLs for plant height with 4.8~19.2% PVE located on LG04.2, LG05.2 and LG06.2 using 94 F_2_ lines^25^. In the present study, a RIL population consisting of 140 lines was evaluated for plant height in three environments. QTL analysis using genotyping and phenotyping data identified a total of 18 QTLs including 8 major QTLs and 10 minor QTLs with 4.85~20.52% PVE. The linkage groups LG04.2, LG05.2 and LG06.2 in the previous study of Shirasawa *et al*.^25^ corresponded to linkage groups A04, B05 and B06 in this study, respectively, by comparing the markers of the two linkage maps. Although 7 QTLs and 5 QTLs were identified on the linkage group A04 and B06, respectively for plant height in this study, there were no repeated markers between these 12 QTLs and above mentioned QTLs in the study of Shirasawa *et al*.^25^. Among these identified QTLs, one QTL, *qPHB4.3b*, was detected on the LG B04 near the marker AHGS2429, which was located on the confidence interval of *qHMSB4* in our previous study for plant height^23^. However, other QTLs were different from those detected in previous studies^23^,^25^, which might be novel QTLs for plant height. In our study, *qPHA4.1c* and *qPHA4.3*, and *qPHA4.1d* and *qPHA4.2b,* respectively overlapped on the linkage group with each other and subsequently were integrated into consensus QTLs *cqPHA4a* and *cqPHA4b*, respectively. Our study detected 8 of 18 QTLs explaining more than 10% PVE, providing insights into genetic controls of plant height in peanut. Nearly one third of missing heritability could be due to limited RIL population size.

In the present study, 8 QTLs with negative additive effects and 10 QTLs with positive additive effects for plant height were identified in the RIL population, implying not all increasing-effect alleles of detected QTLs originated from the high-phenotype parent (ICG 12625) and vice versa. We found that all the lines were recombinants at the 18 detected QTLs, indicating the allelic recombination would be the reason where the RIL population contained nearly a half of lines that expressed as the transgressive segregation of plant height to the parents. This conclusion could be supported by the fact that the tall RILs (from ‘More than ICG 12625’ group) did not harbor majority of the alleles from ICG 12625 but the positive alleles for QTL *per se* (*P* = 2.8E-13). The findings revealed that allelic recombination and favorable-allele pyramiding would be an important way to create the more phenotypic diversity and ultimately improve plant height for achieving a balance of vegetable and productive developments in peanut. In RIL population, either the number of additive alleles or sum of additive effect of the detected QTLs for each RIL were significantly correlated to the observed phenotype of plant height (*P* < 0.05), which suggested that the simple accumulation of QTL alleles allowed to explain the genetic basis of plant height in the peanut RIL population (*R*^2^ = 0.46). It is worth noting that, we detected two consensus QTLs, namely *cqPHA4a* and *cqPHA4b* that stably showed significantly additive effects across three environments. Interestingly, the alleles from the high-phenotype parent (ICG 12625) had an ability to decrease plant height of 5.8~7.67 cm relative to the allele from the low-phenotype parent (Zhonghua 10). These large-effect QTLs provided an effective tool for us to improve plant height of the peanut cultivars by introgression few QTLs/genes via molecular marker-assistant selection.

## Methods

### Plant materials and phenotyping

In this study, a peanut RIL (F_6_ generation) population with 140 lines was developed using the single seed decent procedure at Oil Crops Research Institute (OCRI) of Chinese Academy of Agricultural Sciences (CAAS). Wuhan, China. The maternal parent, Zhonghua 10 (*A. hypogaea* var. *vulgaris*), is a cultivar with middle plant height developed by the OCRI-CAAS, Wuhan, China in 2004. The paternal parent, ICG 12625 (PI497597, *A. hypogaea* var. *aequatoriana*), is a germplasm with high plant height received from the International Crop Research Institute for the Semi-Arid Tropics (ICRISAT), Hyderabad, India. The complete RIL population together with parental lines were planted in experimental field in OCRI-CAAS, Wuhan, China in the consecutive years from 2013 to 2015 using a randomized complete block design with two replications. Each plot contained one row, with 10~12 plants in each row, 10 cm between plants within each row and 30 cm between the rows. Field management followed the standard agricultural practices. At least six plants were selected randomly from each line to investigate the plant height, which measured as the distance from the base of the above-ground plant to the tip of the main stem. Shapiro-Wilk test was used to evaluate the normality of plant height distribution in each year.

With treating the year as a single environment, two-way analysis of variance (ANOVA) was performed to evaluate the effect of genotype and environment on phenotypic variance of plant height in R function “lm”^42^. The line mean based broad-sense heritability for plant height was calculated as:


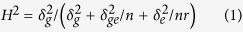


where 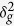 is genetic variance, 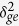 is the variance due to genotype and environment interaction, 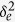 is the residual variance, *n* is the number of environments and r is the number of replications within environment. The estimates of 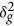, 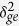 and 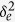 were obtained by the mixed linear model with treating genotype, environment and interaction effect as random effects in the R package “lme4”^42^.

### Genotyping of mapping population and construction of genetic map

Genomic DNA was extracted from young leaves collected from each line using a modified cetyltrimethyl ammonium bromide (CTAB) method. A total of 7,641 SSR and 427 transposon markers were used to screen the polymorphism between two parental lines and the polymorphic markers were then used to genotype the whole RIL population. Markers with prefixes AhTE were transposon markers, which were obtained from the study of Shirasawa *et al*.^25^. The SSR markers with prefixes XY, POCR, AGGS and AHGA were developed by our laboratory^43^,^44^,^45^. Remaining SSR markers with the prefixes pPGPseq, pPGSseq, TC, IPAHM, Ah, RI, EE, EM, GA, GM, GNB, AC, Ad, ARS, gi, AHBGS, PM, AHS, AHGS and HAS were obtained from the literatures^25^,^29^,^32^,^33^,^34^,^35^,^36^,^37^,^39^,^40^. PCR reactions were performed as previously described^24^. For markers detected at more than one polymorphism locus, the loci were named using the suffixes “−1”, “−2” and “-n” after the SSR name, respectively.

The Pearson’s Chi-square test was performed to evaluate the goodness of fit to the expected 1:1 segregation ratio for each locus (*P* < 0.05). The linkage analysis was carried out using JoinMap 3.0^46^ with the minimum logarithm of odds (LOD) of 4.0. The recombinant ratio was transformed into genetic distance by Kossambi map function^47^. Linkage groups were aligned to a published linkage map based on common markers^29^ and the linkage groups (LGs) were designated as A01-A10 and B01-B10 for the A subgenome and B subgenome, respectively.

### QTL, epistasis and meta analyses

QTL analysis was conducted using the composite interval mapping method^48^ in the software windows QTL cartographer 2.5 (http://statgen.ncsu.edu/qtlcart/WQTLCart.htm) and the phenotypic data of plant height obtained during 2013, 2014 and 2015 in Wuhan. The forward regression method based on model 6 (default model) was selected to obtain covariates. The number of control markers, window size and walk space were set to 5, 10 and 2 cM, respectively. LOD threshold value of 2.5 was used to declare the presence of a QTL. In addition, QTL that detected at a LOD value higher than 3.0 and had phenotypic variation explained more than 10% was considered as a major QTL and other QTLs were considered as minor QTLs. We estimated the proportion of variance explained jointly by all identified QTLs based on multiple linear regression using the ‘lm’ function in R software package, which was calculated by comparing the residual sum of squares between the full model and the reduced model excluding identified QTL. The nomenclature of QTL was similar to that described by Udall *et al*.^49^ with the codes 1, 2 and 3 representing for QTL detected in 2013, 2014 and 2015, respectively. QTLs are designated with initial letter *q* followed by the trait name and linkage group. An alphabetical letter was added if two or more QTLs were identified in the same linkage group. For example, if two QTLs for plant height were detected on LG A04, they were named *qPHA4.1a* and *qPHA4.1b*. The identified QTLs were further used to perform epistasis analysis for each environment separately. We firstly used multiple interval mapping (MIM) model implemented in windows QTL cartographer 2.5 to estimate epistasis by default Bayes information content (BIC) criteria. Additionally, a linear regression model including QTL main effect and pair-wise QTL interaction was used to test the significance of all pair-wise QTLs (*P* < 0.05). The proportion of variance explained by each pair-wise QTL was estimated by comparing residual sum of squares between full model and reduced model that excluded interaction term.

QTLs repeatedly detected in different environments and located in the same chromosomal region were subjected to meta-analysis to estimate the position of the underlying meta-QTL^50^. Meta-analysis was performed using the BioMercator 2.1 software^51^. The QTLs repeatedly identified for plant height in different environments were integrated into a consensus QTL, which were then designated with initial letters “*cq*” followed by trait name and linkage groups. An alphabetical letter was added if two or more consensus QTLs were identified on the same linkage group.

## Additional Information

**How to cite this article**: Huang, L. *et al*. Development and deployment of a high-density linkage map identified quantitative trait loci for plant height in peanut (*Arachis hypogaea* L.). *Sci. Rep.* **6**, 39478; doi: 10.1038/srep39478 (2016).

**Publisher's note:** Springer Nature remains neutral with regard to jurisdictional claims in published maps and institutional affiliations.

## Supplementary Material

Supplementary Information File #1

Supplementary Dataset 1

Supplementary Dataset 2

## Figures and Tables

**Table 1:** Phenotypic variation for plant height (cm) in the peanut RILs.

Year	Zhonghua 10	ICG 12625	Mean	SD	CV (%)	Min	Max
2013	42.4	73.9	66.2	16.5	24.9	24.6	125.4
2014	47.3	66.5	66.9	17.8	26.6	29.3	115.7
2015	43.4	62.1	59.4	15.1	25.4	28.2	96.3

**Table 2:** Two-way ANOVA of plant height in the RILs in three environments.

Source	Sum of square	*df*	Mean square	*F* value	*P*
Genotype	158061	139	1137.1	24.1	<0.001
Environment	8876	2	4437.8	93.9	<0.001
Genotype × environment	27840	274	101.6	2.2	<0.001
Error	13801	277	47.2		

**Table 3:** Descriptive statistics of the peanut high-density genetic map in this study.

LGs	Length^a^	Locus^b^	Interval^c^	SDL^d^	Zhonghua 10^e^	ICG 12625^f^
A01	85.39	69 (39)	1.24	4 (5.8)	3	1
A02	86.07	41 (9)	2.10	8 (19.5)	0	8
A03	142.80	89 (25)	1.60	32 (36.0)	31	1
A04	93.12	54 (21)	1.72	1 (1.9)	0	1
A05	108.75	61 (18)	1.78	2 (3.3)	1	1
A06	99.61	29 (5)	3.43	6 (20.7)	6	0
A07	99.69	74 (33)	1.35	5 (6.8)	2	3
A08	130.77	39 (9)	3.35	10 (25.6)	6	4
A09	97.15	76 (23)	1.28	9 (11.8)	8	1
A10	67.61	51 (16)	1.33	3 (5.9)	3	0
B01	118.73	65 (13)	1.83	16 (24.6)	1	15
B02	130.19	71 (24)	1.83	4 (5.6)	4	0
B03	87.41	71 (21)	1.23	2 (2.8)	1	1
B04	121.15	58 (14)	2.09	8 (13.8)	2	6
B05	79.46	59 (20)	1.35	3 (5.1)	2	1
B06	106.87	75 (21)	1.42	7 (9.3)	3	4
B07	136.19	54 (16)	2.52	14 (25.9)	13	1
B08	61.69	45 (13)	1.37	2 (4.4)	1	1
B09	105.44	60 (23)	1.76	6 (10.0)	5	1
B10	80.69	77 (25)	1.05	10 (13.0)	10	0
A subgenome	1010.95	583 (249)	1.73	80 (13.7)	60	20
B subgenome	1027.80	635 (162)	1.62	72 (11.3)	42	30
Whole genome	2038.75	1218 (511)	1.67	152 (12.5)	102	50

^a^Map length for each linkage group (cM); ^b^The number of SSR loci located in each linkage group and common loci mapped on the genetic map (in parentheses); ^c^The average genetic interval between flanking SSR loci (cM); ^d^The number (without the parenthesis) and proportion (within the parenthesis) of segregation distortion loci in each linkage group (*P* < 0.05); ^e^The number of SSR loci that segregated distortedly to the parent ‘Zhonghua 10’; ^f^The number of SSR loci that segregated distortedly to the parent ‘ICG 12625’.

**Table 4:** QTL information of plant height in peanut in three environments.

Environment	LG	QTL	Pos. (cM)	CI (cM)^a^	LOD^b^	A^c^	*R*^2^ (%)^d^	Joint_*R*^2^ (%)^e^
2013	A04	*qPHA4.1a*	54.9	54.6–55.0	6.39	−6.61	15.37	53.3
		*qPHA4.1b*	58.1	57.3–58.5	6.9	−6.93	16.57	
		*qPHA4.1c*	61.9	61.5–62.7	7.98	−7.35	18.7	
		*qPHA4.1d*	70.4	70.1–71.5	7.31	−7.67	20.52	
	A09	*qPHA9.1*	33.7	30.7–34.6	2.84	4.14	6.04	
	B03	*qPHB3.1*	61.9	61.4–62.6	5.21	−6.08	11.24	
2014	A03	*qPHA3.2a*	94.3	93.2–94.8	2.58	3.99	4.85	65.5
		*qPHA3.2b*	103.6	103.4–105.9	4.05	5.1	7.48	
	A04	*qPHA4.2a*	60.6	60.0–60.8	4.9	−5.58	9.45	
		*qPHA4.2b*	69.4	67.0–71.4	4.78	−6.04	10.84	
	B06	*qPHB6.2a*	47	46.1–47.4	2.64	4.28	5	
		*qPHB6.2b*	54.7	54.7–55.6	3.5	4.85	6.6	
		*qPHB6.2c*	64.3	63.7–64.7	3.8	4.99	6.99	
2015	A04	*qPHA4.3*	61.9	61.9–62.6	7.06	−5.8	14.47	62.3
	B04	*qPHB4.3a*	75.4	73.5–76.2	3.3	5.06	10.33	
		*qPHB4.3b*	98.5	97.7–101.1	4.4	4.61	9.24	
	B06	*qPHB6.3a*	67.5	67.2–68.6	3.96	4.3	7.77	
		*qPHB6.3b*	77.7	75.6–78.0	2.59	3.52	5.23	

^a^Confidence interval; ^b^Logarithm of odds; ^c^Additive value; ^d^Phenotypic variance explained by each QTL. ^e^Phenotypic variance explained jointly by all identified QTLs.

**Table 5:** Consensus QTL for plant height obtained by meta-analysis in three environments.

Consensus QTL	LG	Position (cM)	CI (cM)
*cqPHA4a*	A04	61.9	61.6–62.2
*cqPHA4b*	A04	70.3	69.7–71.0
