# Supplementary material for: Development and deployment of a high-density linkage map identified quantitative trait loci for plant height in peanut (Arachis hypogaea L.)
Source: Sci Rep. 2016 Dec 20;6:39478. doi: 10.1038/srep39478 (PMC5171768; doi:10.1038/srep39478)
Supplement: Supplementary Information File #1 [file srep39478-s1.doc]

Supplementary information for:

**Development and deployment of a high-density genetic linkage map identified quantitative trait loci for plant height in peanut (*Arachis hypogaea* L.)**

Li Huang^1^, Xiaoping Ren^1^, Bei Wu^1^, Xinping Li^1^, Weigang Chen^1^, Xiaojing Zhou^1^, Yuning Chen^1^, Manish K Pandey^2^, Yongqing Jiao^1^, Huaiyong Luo^1^, Yong Lei^1^, Rajeev K Varshney^2^, Boshou Liao^1^, Huifang Jiang^1^*

^1^Key Laboratory of Biology and Genetic Improvement of Oil Crops, Ministry of Agriculture, Oil Crops Research Institute of the Chinese Academy of Agricultural Sciences, Wuhan, Hubei, China

^2^International Crops Research Institute for the Semi-Arid Tropics (ICRISAT), Hyderabad, India

* Corresponding author

Email: [peanutlab@oilcrops.cn](mailto:peanutlab@oilcrops.cn)

Tel: 86-27-86711550

Fax: 86-27-86816451

This file includes:

Supplementary Figure 1

Supplementary dataset 1-2

**SUPPLEMENTARY INFORMATION**

**Supplementary Figure 1** Additive distribution of molecular markers across the whole genome.

**Supplementary Dataset 1** Locus position, Primer sequences and segregation distortion for the markers of the linkage map in this study. (Supplementary_Dataset_1.xls).

**Supplementary Dataset 2** Epistatic interaction between pair-wise QTLs for plant height across three environments. (Supplementary_Dataset_2.xls).


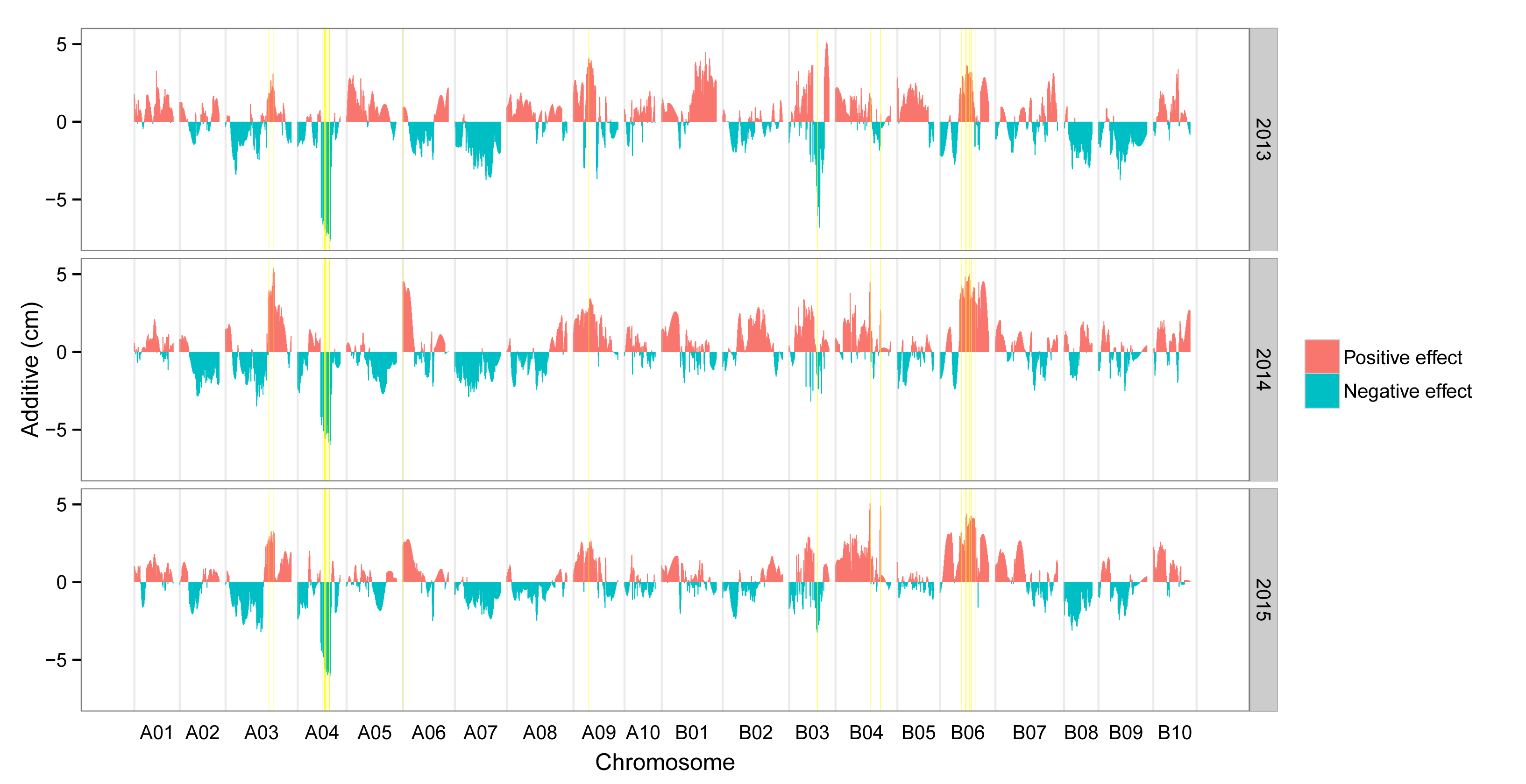


**Supplementary Figure S1.** Additive distribution of molecular markers across the whole genome. The increasing-effect allele of QTL originated from the parent “ICG12615” when the QTL is positively effective, whereas the increasing-effect allele of QTL came from the parent “Zhonghua10” when the QTL is negatively effective.
